# Development and validation of a nomogram for predicting the overall survival of patients with testicular cancer

**DOI:** 10.1002/cam4.6203

**Published:** 2023-06-01

**Authors:** Haohui Yu, Bin Feng, Yunrui Zhang, Jun Lyu

**Affiliations:** ^1^ Department of Medical Administration The First Affiliated Hospital of Jinan University Guangzhou China

**Keywords:** nomogram, overall survival, SEER, testicular cancer

## Abstract

**Background:**

The purpose of this study was to develop and validate a nomogram to predict survival in testicular cancer patients.

**Methods:**

Testicular cancer patients diagnosed between 2004 and 2015 from the Surveillance, Epidemiology, and End Results (SEER) database were selected for this study. A random sampling method was used to divide patients into training and validation cohorts, which accounted for 30% and 70% of the total sample, respectively. The nomogram was developed using the training cohort and evaluated using the C index, calibration chart, and area under the receiver operating characteristic curve (AUC).

**Results:**

Seven risk factors that affect the survival of testicular cancer patients (AJCC stage, marital status, age at diagnosis, race, SEER historic stage A, surgery status, and origin) were identified using Cox proportional hazard regression analysis. The nomogram has a higher C index (0.897) and AUC when compared with the AJCC staging system. The results of the calibration chart of the nomogram show that the predicted survival of testicular cancer patients at 3, 5, and 10 years after diagnosis is very close to their actual survival.

**Conclusions:**

We developed and validated a nomogram for predicting the survival rate of testicular cancer patients at 3, 5, and 10 years after diagnosis. This nomogram has better discrimination, calibration, and clinical validity than the AJCC staging system. This indicates that the nomogram can be used to predict the survival of testicular cancer patients effectively, and provide a reference for patient treatment strategies.

## 1 BACKGROUND

Testicular cancer accounts for about 0.4% of all cancers.^1^, ^2^ There were about 71,000 new testicular cancer patients and 9500 deaths globally in 2018, mostly in developed regions such as Europe, Australia, and North America.^2^ There are approximately 9300 new testicular cancer patients in the US every year, making it the 18th most common tumor in males.^3^, ^4^ Testicular cancer is located in the male reproductive system, and is the second most common after prostate cancer.^5^, ^6^ Testicular cancer is most prevalent in males younger than 45 years, and mainly consists of germ cell tumors (more than 90% of cases).^7^, ^8^, ^9^

Testicular cancer is a disease caused by multiple factors, such as genetics, diet, and lifestyle.^10^, ^11^, ^12^ The incidence of testicular cancer reportedly differs between ethnic groups, being highest in whites.^13^, ^14^ Cryptorchidism is also a risk factor for testicular cancer.^15^ Cryptorchidism patients have an incidence 10 times that of other patients.^16^ Testicular cancer is mainly treated through surgical removal of the tumor combined with radiotherapy and chemotherapy, with most patients having a good prognosis.^17^ However, some patients respond poorly to the treatment.

At present, the TNM staging system is often used to predict the survival outcome of testicular cancer patients.^18^ However, the TNM staging system only accounts for the impact of some tumor characteristics such as size, invasion status, and metastasis on the survival of patients, and does not consider individual differences such as sex, age, and race for its prognosis.^19^ With the discovery of additional cancer risk factors and scientific and technological developments, it is necessary to develop a new method for predicting patient survival that includes tumor characteristics, demographic, and treatment methods. A nomogram is a graphical representation of a model that combines a variety of risk factors, and has been widely used to predict the overall survival of different types of cancer patients.^19^, ^20^

This study explored risk factors that potentially affect the prognosis of testicular cancer patients based on the Surveillance, Epidemiology, and End Results (SEER) 18 database, and developed a nomogram to predict their overall survival.

## 2 METHODS

### 2.1 Data source

In this study, the data of testicular cancer patients between 2004 and 2015 were extracted from the SEER 18 database. The SEER database covers approximately 30% of the US population and contains a large amount of cancer research data. The SEER 18 database collected data related to various patient tumors between 1973 and 2015, including demographics, tumor characteristics, treatment methods, and survival data. Signing the SEER Research Data Agreement provided us with access to the SEER database.

### 2.2 Study population and inclusion criteria

The SEER*Stat software (version 8.3.6) was used to identify testicular cancer data from the SEER 18 database using its code from the International Taxonomy of Tumors (third edition). The main site codes of testicular cancer are C62.0–C62.9, and are classified according to location, including undescended testis (C62.0), descended testis (C62.1), and testis (C62.9). Testicular cancer patients with incomplete data on race, AJCC stage, and survival, as well as those younger than 20 years were excluded. We extracted 25,468 testicular cancer patients from the SEER 18 database, who were divided into a training cohort (70%) and a validation cohort (30%) using random sampling. The patient screening process is shown in Figure S1 in the Supplement. The data collected from all patients included race, age at diagnosis, AJCC stage, tumor size, year of diagnosis, surgery status, chemotherapy status, radiotherapy status, marital status, survival time, and tumor extension.

### 2.3 Statistical analysis

We used univariable and multivariable Cox proportional hazard regression analyses to calculate the hazard ratio (HR) and 95% confidence interval (CI) of each risk factor for testicular cancer. The Akaike Information Criteria (AIC) was used as the selection criteria, and selected the variable with the lowest AIC in the model as the final predictor variable. Random sampling was used to divide patients into training and validation cohorts, of which the training cohort accounts for 70% of the sample. By screening the significant predictor variables, we used the training cohort to construct a nomogram and predict the survival of each patient 3, 5, and 10 years after diagnosis. The performance of the nomogram was internally compared with the C index of the AJCC staging system (sixth edition), and the accuracy of the nomogram was identified using 1000 iterations of bootstrap resampling. We evaluated the accuracy of the 3‐, 5‐ and 10‐year survival predictions using the area under the receiver operating characteristic curve (AUC), and evaluated the performance of the nomogram using calibration graphs. The same method was applied to the validation cohort to validate the nomogram. The accuracy of the nomogram was compared with that of the AJCC staging system through the net reclassification improvement (NRI) and the integrated discrimination improvement (IDI) values. The clinical validity of the nomogram was evaluated by decision‐curve analysis (DCA). All statistical analyses were performed using R software (version 3.6.3). The significance level was set to *p* < 0.05.

## 3 RESULTS

### 3.1 Basic characteristics of patients

This study included 25,468 testicular cancer patients older than 20 years who were diagnosed between 2004 and 2015. The patients were randomly divided into the training cohort (17,827 patients) and the validation cohort (7641 patients). The patients were aged 37.2 ± 13.0 and 37.0 ± 12.9 years (mean ± SD) in the training and validation cohorts, respectively, and were mostly White (91.6% and 92.5%), at AJCC stage I (73.2% and 73.4%), and at the localized stage (66.2% and 66.4%). The basic characteristics of the patients are listed in Table 1.

**TABLE 1:** The basic characteristics of testicular cancer patients.

Variables	Overall, *N* (%)	Training set, *N* (%)	Validating set, *N* (%)
Patients	25,468	17,827 (70.0)	7641 (30.0)
Age at diagnosis			
Mean ± SD (years)	37.1 ± 13.0	37.2 ± 13.0	37.0 ± 12.9
Marital			
Married	11,766 (46.2)	8277 (46.4)	3489 (45.7)
Unmarried	13,702 (53.8)	9550 (53.6)	4152 (54.3)
AJCC			
I	18,662 (73.3)	13,055 (73.2)	5607 (73.4)
II	3014 (11.8)	2107 (11.8)	907 (11.9)
III	3607 (14.2)	2538 (14.2)	1069 (14.0)
IV	185 (0.7)	127 (0.8)	58 (0.8)
SEER historic stage A			
Distant	2992 (11.7)	2116 (11.9)	876 (11.5)
Localized	16,879 (66.3)	11,804 (66.2)	5075 (66.4)
Regional	4607 (18.1)	3208 (18.0)	1399 (18.3)
Unstaged	990 (3.9)	699 (3.9)	291 (3.8)
Surgery			
No	659 (2.6)	460 (2.6)	199 (2.6)
Yes	24,809 (97.4)	17,367 (97.4)	7442 (97.4)
Race			
Black	758 (3.0)	537 (3.0)	221 (2.9)
Other^a^	1312 (5.2)	960 (5.4)	352 (4.6)
White	23,398 (91.9)	16,330 (91.6)	7068 (92.5)
Origin			
Non‐Spanish‐Hispanic‐Latino	20,170 (79.2)	14,205 (79.7)	5965 (78.1)
Spanish‐Hispanic‐Latino	5298 (20.8)	3622 (20.3)	1676 (21.9)
Site			
Undescended testis	426 (1.7)	311 (1.7)	115 (1.5)
Descended testis	11,227 (44.1)	7872 (44.2)	3355 (43.9)
Testis	13,815 (54.2)	9644 (54.1)	3356 (54.6)
T			
T0	263 (1.1)	190 (1.1)	73 (1.0)
T1	15,654 (63.9)	10,930 (63.8)	4724 (64.3)
T2	6392 (26.1)	4461 (26.0)	1931 (26.3)
T3	1132 (4.6)	801 (4.7)	331 (4.5)
T4	161 (0.7)	118 (0.7)	43 (0.6)
TX	881 (3.6)	632 (3.7)	249 (3.4)
N			
N0	19,031 (77.7)	13,323 (77.8)	5708 (77.6)
N1	2533 (10.3)	1774 (10.4)	759 (10.3)
N2	1364 (5.6)	943 (5.5)	421 (5.7)
N3	1256 (5.1)	871 (5.1)	385 (5.2)
NX	299 (1.2)	221 (1.3)	78 (1.1)
M			
M0	21,504 (87.8)	15,032 (87.7)	6472 (88.0)
M1a	1705 (7.0)	1207 (7.0)	498 (6.8)
M1b	1148 (4.7)	809 (4.7)	339 (4.6)
M1NOS	104 (0.4)	72 (0.4)	32 (0.4)
MX	22 (0.1)	12 (0.1)	10 (0.1)
Radiation			
No	20,101 (78.9)	14,076 (79.0)	6025 (78.9)
Yes	5367 (21.1)	3751 (21.0)	1616 (21.1)
Chemotherapy			
No	16,107 (63.2)	11,292 (63.3)	4815 (63.0)
Yes	9361 (36.8)	6535 (36.7)	2826 (37.0)
Region			
Alaska	43 (0.2)	31 (0.2)	12 (0.2)
East	7994 (31.4)	5566 (31.2)	2428 (31.8)
Northern Plains	2143 (8.4)	1508 (8.5)	635 (8.3)
Pacific Coast	13,637 (53.5)	9546 (53.5)	4091 (53.5)
Southwest	1651 (6.5)	1176 (6.6)	475 (6.2)
State			
Alaska	43 (0.2)	31 (0.2)	12 (0.2)
California	11,380 (44.7)	7980 (44.8)	3400 (44.5)
Connecticut	1155 (4.5)	791 (4.4)	364 (4.8)
Georgia	2057 (8.1)	1423 (8.0)	634 (8.3)
Hawaii	390 (1.5)	272 (1.5)	118 (1.5)
Iowa	998 (3.9)	722 (4.1)	276 (3.6)
Kentucky	1203 (4.7)	846 (4.7)	357 (4.7)
Louisiana	1042 (4.1)	712 (4.0)	330 (4.3)
Michigan	1145 (4.5)	786 (4.4)	359 (4.7)
New Jersey	2537 (10.0)	1794 (10.1)	743 (9.7)
New Mexico	617 (2.4)	437 (2.5)	180 (2.4)
Utah	1034 (4.1)	739 (4.1)	295 (3.9)
Washington	1867 (7.3)	1294 (7.3)	573 (7.5)
Cause‐specific death			
Alive	24,382 (95.7)	17,016 (95.5)	7366 (96.4)
Dead	1086 (4.3)	811 (4.5)	275 (3.6)
Survival time			
Median (month)	58.0 (23.0, 97.0)	57.0 (23.0, 97.0)	58.0 (23.0, 97.0)
Tumor size			
Median (mm)	42.0 (25.0, 70.0)	42.0 (25.0, 70.0)	42.0 (25.0, 70.0)
Tumor extension			
Median (mm)	16.0 (10.0, 30.0)	16.0 (10.0, 30.0)	16.0 (10.0, 30.0)

Abbreviation: SEER, Surveillance, Epidemiology, and End Results.

^a^ Other refers to American Indian/AK Native, Asian/Pacific Islander.

### 3.2 Screening of model survival predictors

We screened all 25,468 patients included in this study and selected 17 possible risk factors, which included race, age at diagnosis, cancer site, year of diagnosis, origin, marital status, SEER historic stage A, state, region, AJCC stage, tumor size, tumor extension, surgery status, radiotherapy status, and chemotherapy status (Table 1). Variables related to the survival of testicular cancer patients were screened using univariable and multivariable Cox proportional hazards regression analysis. The variables included in the model as the final predictors were selected when the model's AIC was the lowest. We identified the seven predictors most closely related to survival (*p* < 0.05), which were age at diagnosis (HR = 1.03, 95% CI = 1.02–1.03, *p* < 0.001), marital status (unmarried vs. married: HR = 1.95, 95% CI = 1.66–2.28, *p* < 0.001), AJCC stage (III vs. I: HR = 2.44, 95% CI = 1.37–4.36, *p* = 0.002; IV vs. I: HR = 4.27, 95% CI = 3.48–6.31, *p* < 0.001), SEER historic stage A (localized vs. distant: HR = 0.23, 95% CI = 0.12–0.45, *p* < 0.001; regional vs. distant: HR = 0.56, 95% CI = 0.33–0.94, *p* = 0.029), surgery status (yes vs. no: HR = 0.48, 95% CI = 0.37–0.63, *p* < 0.001), race (White vs. Black: HR = 0.65, 95% CI = 0.48–0.87, *p* = 0.004), and origin (Spanish‐Hispanic‐Latino vs. non‐Spanish‐Hispanic‐Latino: HR = 1.35, 95% CI = 1.14–1.60, *p* < 0.001). The HRs and 95% CIs of the factors related to the survival of testicular cancer patients are listed in Table 2.

**TABLE 2:** Univariable and multivariable Cox proportional hazard regression analysis of testicular cancer patients.

Variables	Univariable analysis		Multivariable analysis	
	HR (95% CI)	*p*‐Value	HR (95% CI)	*p*‐Value
Age at diagnosis	1.04 (1.03–1.04)	<0.001***	1.03 (1.02–1.03)	<0.001***
Marital				
Married	Reference		Reference	
Unmarried	1.68 (1.48–1.90)	<0.001***	1.95 (1.66–2.28)	<0.001***
AJCC				
I	Reference		Reference	
II	2.74 (2.16–3.47)	<0.001***	1.05 (0.63–1.76)	0.849
III	17.68 (15.22–20.54)	<0.001***	2.44 (1.37–4.36)	0.002**
IV	50.96 (39.48–65.77)	<0.001***	4.27 (3.48–6.31)	<0.001***
SEER historic stage A				
Distant	Reference		Reference	
Localized	0.03 (0.02–0.03)	<0.001***	0.23 (0.12–0.45)	<0.001***
Regional	0.10 (0.08–0.13)	<0.001***	0.56 (0.33–0.94)	0.029*
Unstaged	1.12 (0.97–1.31)	0.130	3.03 (0.38–24.15)	0.295
Surgery				
No	Reference		Reference	
Yes	0.08 (0.07–0.09)	<0.001***	0.48 (0.37–0.63)	<0.001***
Race				
Black	Reference		Reference	
Other^a^	0.73 (0.52–1.02)	0.068	0.83 (0.55–1.25)	0.369
White	0.49 (0.38–0.64)	<0.001***	0.65 (0.48–0.87)	0.004**
Origin				
Non‐Spanish‐Hispanic‐Latino	Reference		Reference	
Spanish‐Hispanic‐Latino	1.48 (1.30–1.70)	<0.001***	1.35 (1.14–1.60)	<0.001***
Site				
C62.0‐undescended testis	Reference		Reference	
C62.1‐descended testis	1.07 (0.66–1.74)	0.776	1.72 (0.93–2.91)	0.072
C62.9‐testis, NOS	1.11 (0.68–1.79)	0.678	1.70 (0.97–2.85)	0.067
T				
T0	Reference		Reference	
T1	0.10 (0.07–0.14)	<0.001***	1.13 (0.69–1.85)	0.632
T2	0.14 (0.1–0.2)	<0.001***	1.13 (0.69–1.85)	0.623
T3	0.50 (0.35–0.72)	<0.001***	1.28 (0.82–2)	0.282
T4	1.72 (1.12–2.62)	0.012*	2.00 (0.92–3.18)	0.071
TX	1.40 (1.01–1.94)	0.043*	1.26 (0.86–1.85)	0.231
N				
N0	Reference		Reference	
N1	5.54 (4.69–6.54)	<0.001***	1.21 (0.99–1.48)	0.061
N2	2.69 (2.05–3.53)	<0.001***	0.79 (0.59–1.07)	0.123
N3	6.99 (5.75–8.51)	<0.001***	1.16 (0.93–1.46)	0.186
NX	19.95 (15.76–25.26)	<0.001***	1.05 (0.8–1.37)	0.721
M				
M0	Reference		Reference	
M1a	14.76 (12.33–17.66)	<0.001***	1.75 (0.95–3.25)	0.074
M1b	38.10 (32.3–44.94)	<0.001***	2.73 (0.98–6)	0.069
M1NOS	18.69 (11.71–29.83)	<0.001***	1.53 (0.71–3.31)	0.281
MX	8.66 (2.15–34.84)	0.002**	2.25 (0.53–9.49)	0.270
Tumor size	1.00 (1.00–1.00)	<0.001***	1.00 (1.00–1.00)	0.280
Tumor extension	1.00 (1.00–1.00)	<0.001***	1.00 (1.00–1.00)	0.980
Radiation				
No	Reference		Reference	
Yes	0.66 (0.56–0.77)	0.000	1.27 (1.04–1.54)	0.020
Chemotherapy				
No	Reference		Reference	
Yes	6.03 (5.24–6.93)	0.000	0.97 (0.78–1.2)	0.760
Region				
Alaska	Reference		Reference	
East	0.88 (0.22–3.54)	0.858	0.33 (0.08–1.42)	0.138
Northern Plains	0.83 (0.20–3.35)	0.789	0.44 (0.1–1.89)	0.267
Pacific Coast	0.96 (0.24–3.85)	0.954	0.25 (0.06–1.09)	0.065
Southwest	0.70 (0.17–2.85)	0.614	0.40 (0.09–1.75)	0.223
State				
Alaska	Reference		Reference	
California	1.07 (0.27–4.28)	0.925	1.48 (0.48–3.31)	0.425
Connecticut	0.45 (0.11–1.90)	0.276	0.73 (0.42–1.26)	0.262
Georgia	1.09 (0.27–4.41)	0.906	1.53 (0.89–2.15)	0.114
Hawaii	0.60 (0.13–2.69)	0.502	1.09 (0.47–2.55)	0.843
Iowa	0.80 (0.19–3.30)	0.755	1.05 (0.64–1.74)	0.840
Kentucky	1.10 (0.27–4.50)	0.894	1.94 (0.75–2.87)	0.421
Louisiana	1.31 (0.32–5.36)	0.705	1.86 (0.52–4.09)	0.675
Michigan	0.85 (0.21–3.50)	0.822	1.36 (0.67–4.23)	0.623
New Jersey	0.64 (0.16–2.60)	0.533	1.73 (0.91–3.14)	0.716
New Mexico	1.11 (0.27–4.62)	0.890	1.20 (0.66–2.17)	0.546
Utah	0.46 (0.11–1.94)	0.290	0.83 (0.47–2.81)	0.472
Washington	0.41 (0.1–1.72)	0.224	0.96 (0.63–2.41)	0.714

Abbreviations: CI, confidence interval; HR, hazard ratio; SEER, Surveillance, Epidemiology, and End Results.

^a^ Other refers to American Indian/AK Native, Asian/Pacific Islander.

* *p* < 0.05

** *p* < 0.01

*** *p* < 0.001.

### 3.3 Survival curves

To further analyze the impact of the selected predictors on the survival of testicular cancer patients, we performed a Cox survival regression analysis on the patient data. As shown in Figure S2 in the Supplement, the high‐risk factors that affected the survival of testicular cancer patients were being unmarried (Figure S2A), in the late AJCC stages (Figure S2B), in the distant stage (Figure S2C), without surgery (Figure S2D), Black (Figure S2E) and Spanish‐Hispanic‐Latino (Figure S2F).

### 3.4 Nomogram development

**FIGURE 1:**
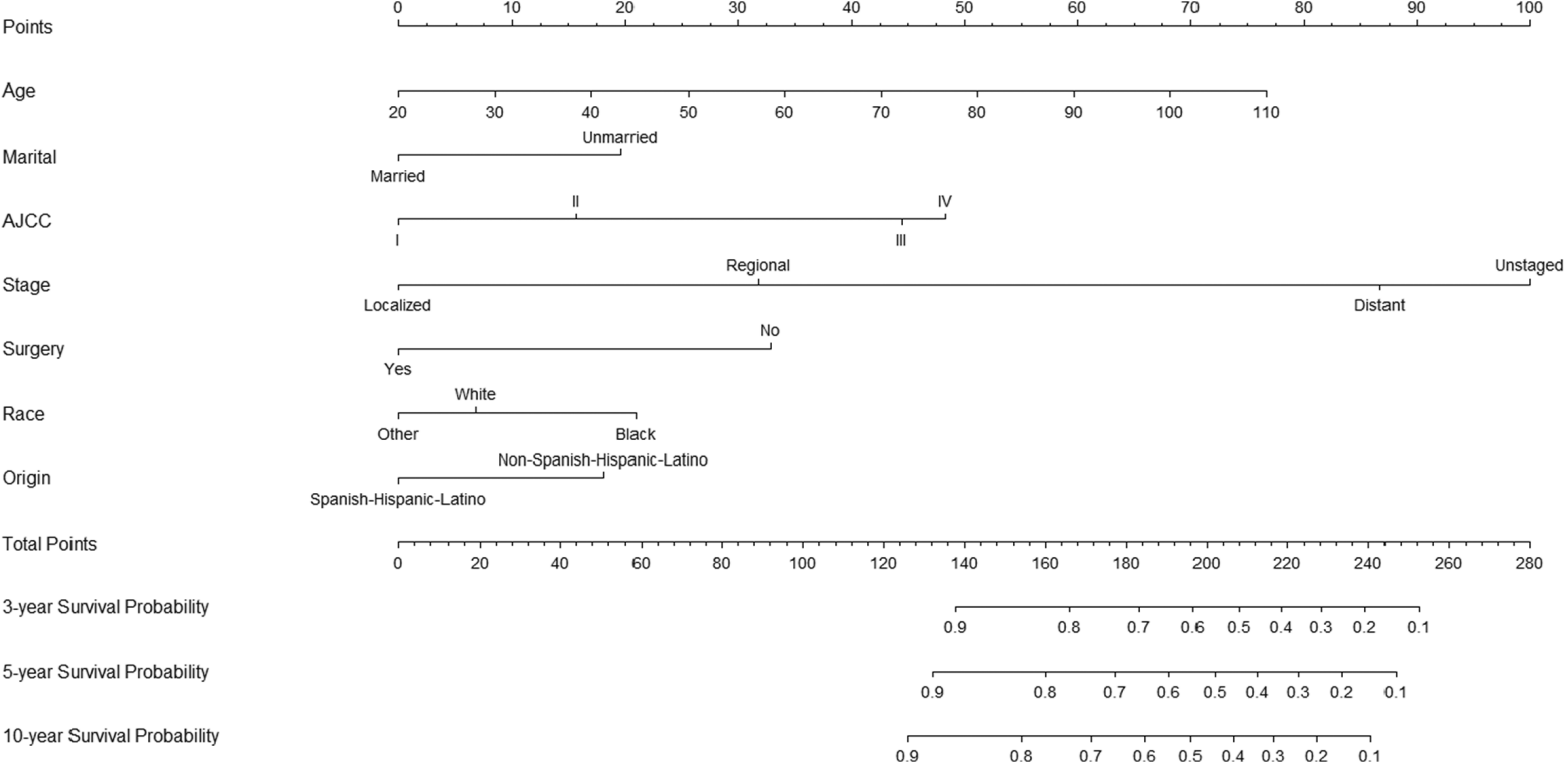
Nomogram predicting the 3‐, 5‐, and 10‐year overall survival of testicular cancer patients. Using the nomogram, we determine the position of each variable of the patient on the corresponding axis, and calculate the corresponding score on the points axis. Then calculate the total score of all variables of the patient. Finally, draw a straight line on the score corresponding to the total points axis to determine the patient's survival rate at 3‐, 5‐, and 10‐year.

According to the seven screened predictors, we used the training cohort to construct the survival prediction nomogram of testicular cancer patients, which had a C index of 0.898. Based on the nomogram, we calculated the overall survival of testicular cancer patients at 3, 5, and 10 years after diagnosis (Figure 1). According to the characteristics of each testicular cancer patient, we can calculate each predictor's score and calculate the survival of the patient in 3, 5, and 10 years after survival based on the total score of all predictors. We compared the resolution of the nomogram and the AJCC staging system through 1000 iterations of bootstrap resampling. We found that the C index was higher for the nomogram (0.898) than for the AJCC staging system (0.834).

**FIGURE 2:**
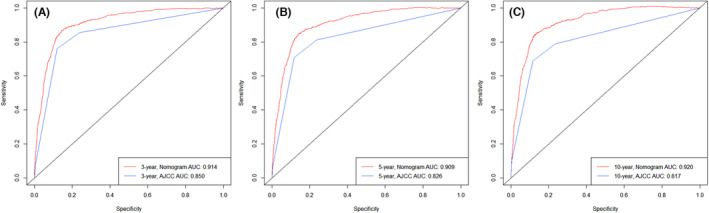
Comparison of the receiver operating characteristic curve (AUC) of nomogram and AJCC staging system in the training set. The AUC is used to predict the overall survival of testicular cancer patients at 3‐year (A), 5‐year (B), and 10‐year (C). The red line represents the overall survival predicted by the nomogram, and the blue line represents the overall survival predicted by AJCC staging system.

In addition, we compared the AUCs when using the nomogram and the AJCC staging system to predict the overall survival of testicular cancer patients 3, 5, and 10 years after diagnosis. For the nomogram, the AUCs predicting the overall survival of the patient at 3, 5, and 10 years after diagnosis were 0.914 (Figure 2A), 0.909 (Figure 2B), and 0.920 (Figure 2C), respectively. The AJCC staging system predicted that the AUCs of the overall survival at 3, 5, and 10 years after diagnosis were 0.850 (Figure 2A), 0.826 (Figure 2B), and 0.817 (Figure 2C), respectively. The AUCs of the nomogram and the AJCC staging system for the training cohort are shown in Figure 2.

**FIGURE 3:**
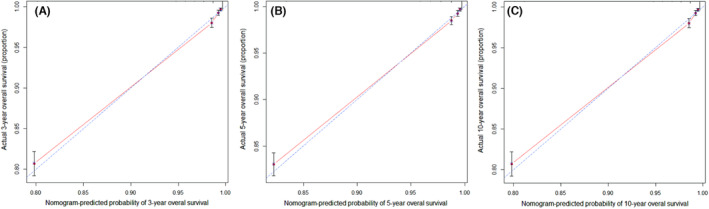
The calibration of the nomogram using the training set. The x‐axis represents the overall survival predicted by the nomogram, and the y‐axis represents the actual survival. (A) 3‐year overall survival. (B) 5‐year overall survival. (C) 10‐year overall survival.

Finally, we constructed calibration charts for the nomogram based on patients in the training cohort at 3, 5, and 10 years after diagnosis to verify the similarity between the survival predicted by the nomogram and the actual survival of patients (Figure 3). The results show that the nomogram's 3‐, 5‐ and 10‐year survival predictions were very close to the actual survival.

### 3.5 Nomogram validation

**FIGURE 4:**
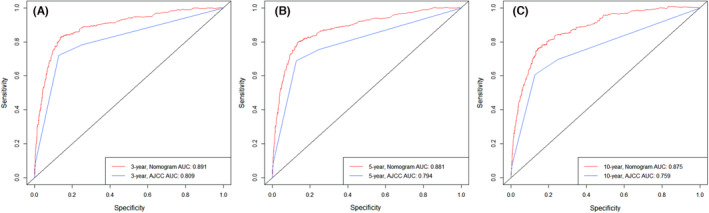
Comparison of the receiver operating characteristic curve (AUC) of nomogram and AJCC staging system in the validation set. The AUC is used to predict the overall survival of testicular cancer patients at 3‐year (A), 5‐year (B), and 10‐year (C). The red line represents the overall survival predicted by the nomogram, and the blue line represents the overall survival predicted by AJCC staging system.

We used the validation cohort data to verify the nomogram, using the same method we used to construct the model. We compared the resolution of the nomogram and the AJCC staging system in the validation cohort through 1000 iterations of bootstrap resampling. The results show that the nomogram for the validation cohort has a higher C index (0.872) than the AJCC staging system (0.797). We then compared the AUC in the validation cohort for both models to predict the overall survival of testicular cancer patients at 3, 5, and 10 years after diagnosis. In the validation cohort, the AUCs predicting the overall survival of patients at 3, 5, and 10 years after diagnosis (see Figure 4A–C, respectively) were 0.891, 0.881, and 0.875, respectively, in the nomogram, and 0.809, 0.794, and 0.759 in the AJCC staging system. The AUC of the validation cohort from the nomogram and AJCC staging system are shown in Figure 4.

**FIGURE 5:**
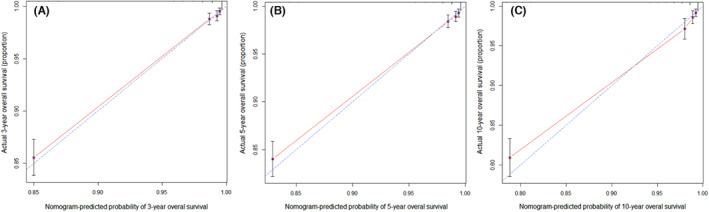
The calibration of the nomogram using the validation set. The x‐axis represents the overall survival predicted by the nomogram, and the y‐axis represents the actual survival. (A) 3‐year overall survival. (B) 5‐year overall survival. (C) 10‐year overall survival.

We constructed calibration plots on the nomogram for patients in the validation cohort 3, 5, and 10 years after diagnosis. The results show that the 3‐, 5‐, and 10‐year survival predictions by the nomogram are very close to the actual survival rates (Figure 5).

**FIGURE 6:**
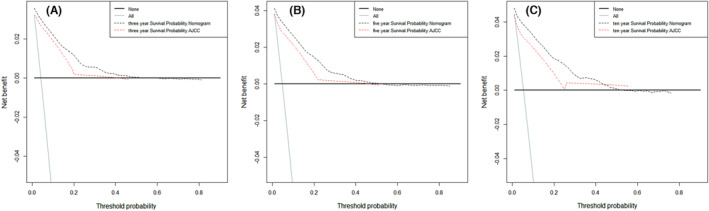
The decision curve analysis of the nomogram and the AJCC staging system in the validation set. The decision curve analysis for predicting the prognosis of testicular cancer patients atn 3‐year (A), 5‐year (B), and 10‐year (C). The black dotted line represents the nomogram, and the red dotted line represents the AJCC staging system.

Finally, we compared the accuracy of the nomogram with that of the AJCC staging system for the validation cohort. At 3, 5, and 10 years after diagnosis, the NRI values were 0.379 (95% CI = 0.276–0.491), 0.383 (95% CI = 0.263–0.489), and 0.422 (95% CI = 0.307–0.507), respectively, while the IDI values were 0.066 (*p* < 0.001), 0.078 (*p* < 0.001), and 0.088 (*p* < 0.001). The DCA curves of the nomogram for the validation cohort at 3, 5, and 10 years after diagnosis are shown in Figure 6. The results show that the nomogram is more clinically effective and accurate at predicting the survival of testicular cancer patients than the AJCC staging system.

## 4 DISCUSSION

Testicular cancer is a cancer of the reproductive system that is common in young males and is the 29th most common new type of cancer in the world.^2^ There are approximately 9300 new testicular cancer patients in the US each year, making it the 18th most common new cancer for male patients.^4^ Testicular cancers mostly consist of germ cell tumors, which often have a serious impact on health.^21^, ^22^ Therefore, early detection and treatment are of great importance in improving therapeutic effects and prolonging survival of patients with testicular cancer.^17^ At present, the AJCC staging system is the most common tool used by clinicians in predicting the survival of cancer patients. It cannot predict the survival of the individual as it only contains the relevant characteristics of the tumor.^23^ Therefore, the development of a new method that individualizes survival predictions is of great importance for testicular cancer patients.

Due to the limitations of the AJCC staging system, nomograms have become a new method for predicting the overall survival of cancer patients in recent years.^19^ A nomogram is a predictive model that includes a variety of predictors, such as tumor and demographic characteristics, and types of therapy.^24^, ^25^, ^26^ It displays the predicted survival of patients in a graphical manner based on a complex mathematical formula. Using the nomogram, we can calculate the score of each predictor variable and its cumulative score that matches with the results list to predict the survival of each patient.^27^, ^28^ Given that nomograms can contain diverse predictors and provide accurate predictions, they have been widely used to predict the survival of many other cancers, such as lung, breast, liver, stomach, and prostate cancer.^29^, ^30^, ^31^, ^32^

The present study constructed a nomogram for predicting the survival of testicular cancer patients. We first extracted data on 25,468 testicular cancer patients from the SEER database, and analyzed the risk factors that affect their survival using multivariable COX regression analysis. We identified the seven predictors most relevant to survival (*p* < 0.05) based on the AIC criteria, which were AJCC stage, race, SEER historic stage A, age at diagnosis, surgery status, marital status, and origin. We next clarified the impact of these factors on the long‐term survival of testicular cancer patients using multivariable COX regression analysis. Therefore, we decided to include these in the final forecast nomogram. We then constructed a nomogram for the training cohort based on the filtered predictors. The nomogram for the training cohort has a higher C index (0.898) than the AJCC staging system (0.834). The nomogram has a higher AUC than the AJCC staging system for 3, 5, and 10 years after diagnosis. According to the results of the calibration curve of the training cohort at 3, 5, and 10 years after diagnosis, we found that the nomogram's predictions on testicular cancer survival are very close to the actual survival. This indicates that the nomogram is more accurate in predicting the survival of testicular cancer patients than the AJCC staging system.

We used the validation cohort to validate the nomogram for testicular cancer patient survival. The C index of the nomogram for the validation cohort (0.872) was similar to that for the training cohort, but higher than that of the AJCC staging system (0.797). The AUC values of the validation cohort for 3, 5, and 10 years after diagnosis are similar to the nomogram of the training cohort. This indicates that the nomogram for the validation cohort has similar testicular cancer survival predictions to the nomogram of the training cohort. Meanwhile, we constructed calibration curves for the validation cohort at 3, 5, and 10 years after diagnosis, which confirmed this conclusion. We then evaluated the clinical significance of the nomogram for predicting the survival of testicular cancer patients. We found that the nomogram had higher NRI and IDI values for 3, 5, and 10 years after diagnosis than the AJCC staging system. This indicates that the nomogram has more accurate predictions for the overall survival of testicular cancer patients. DCA is often considered to be useful for verifying the benefits and clinical validity of a model.^19^, ^33^, ^34^ In our research, the nomogram had better DCA results than the AJCC staging system at 3, 5, and 10 years after diagnosis. This indicates that, compared to the AJCC staging system, the nomogram is more clinically effective and accurate in predicting the survival of testicular cancer patients. In summary, the nomogram we constructed is better than the AJCC staging system at predicting the survival of testicular cancer patients, and provides a reference for patient treatment strategies.

Our study had several limitations. First, the research data comes from the SEER database which lacks some information, such as basic disease status, education level, drug treatments, religious beliefs, and family history, which may have an impact on the survival of testicular cancer patients. Second, cohort studies have inherent limitations, such as possible selection and information bias. Third, there are inherent limitations for any nomogram, such as the assumption that the data collected and analyzed are static in time, and there are no recognized reporting standards for performance.^19^ In addition, our study only included testicular cancer patients in some regions of the US, therefore external data verification needs to be added for it to be applied to other regions. Future studies should include testicular cancer patients from other countries or regions to further verify the nomogram.

## 5 CONCLUSIONS

We screened and identified seven predictors that were most relevant to the survival of testicular cancer patients, which were AJCC stage, race, SEER historic stage A, age at diagnosis, surgery status, marital status, and origin. We developed and validated a nomogram based on these predictors, and used it to predict the survival rate of testicular cancer patients at 3, 5, and 10 years after diagnosis. The nomogram we constructed has improved discrimination, calibration, and clinical validity compared to the AJCC staging system. This indicates that the nomogram can be used to predict the overall survival of testicular cancer patients accurately, and can provide a reference for patient treatment strategies.

## AUTHOR CONTRIBUTIONS

**Haohui Yu:** Data curation (equal); formal analysis (equal); methodology (equal); visualization (equal); writing – original draft (equal). **Bin Feng:** Data curation (equal); methodology (equal); visualization (equal). **Yunrui Zhang:** Data curation (equal); visualization (equal). **Jun Lyu:** Conceptualization (equal); funding acquisition (equal); methodology (equal); project administration (equal).

## FUNDING INFORMATION

None.

## CONFLICT OF INTEREST STATEMENT

None.

## ETHICS STATEMENT

The SEER database is a tumor‐related database developed by the National Cancer Institute of the United States, providing research data for researchers free of charge. All patients participating in the study received the ethical approval sought by the National Cancer Institute.

## CONSENT FOR PUBLICATION

Consent for publication was obtained from all participants.

## Supporting information


Figure S1.
Figure S2.

## Data Availability

We obtained permission to access the database after signing and submitting the SEER Research Data Agreement form via email. The data that support the findings of this study are available from SEER database but restrictions apply to the availability of these data, which were used under license for the current study, and so are not publicly available. Data are however available from the authors upon reasonable request and with permission of SEER database.
