# Supplementary material for: Development and validation of a nomogram for predicting the overall survival of patients with testicular cancer
Source: Cancer Med. 2023 Jun 1;12(14):15567–78. doi: 10.1002/cam4.6203 (PMC10417196; doi:10.1002/cam4.6203)
Supplement: Supplementary file 1 — Figure S1. Figure S2. [file CAM4-12-15567-s001.docx]

**Development and Validation of a Nomogram for predicting the Overall Survival of Patients With Testicular Cancer**

**Supplement Online**

**Figure legends**

Figure S1. The flow diagram of testicular cancer patients selection.

Figure S2. Comparison chart of survival curves of testicular cancer patients.


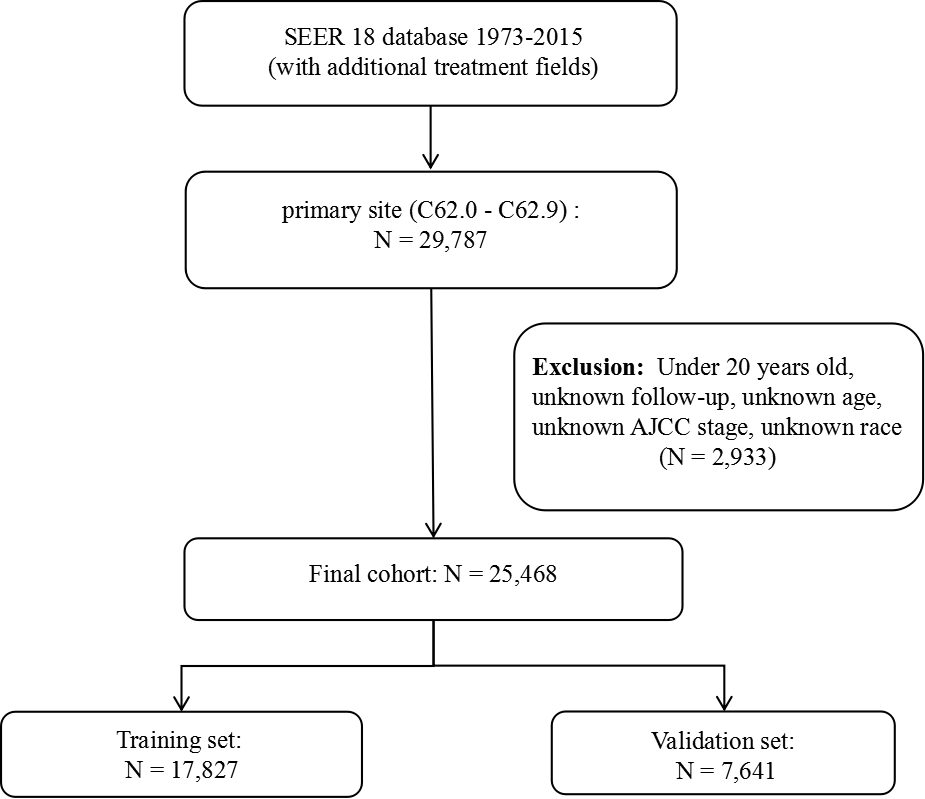


eFigure 1. The flow diagram of testicular cancer patients selection.


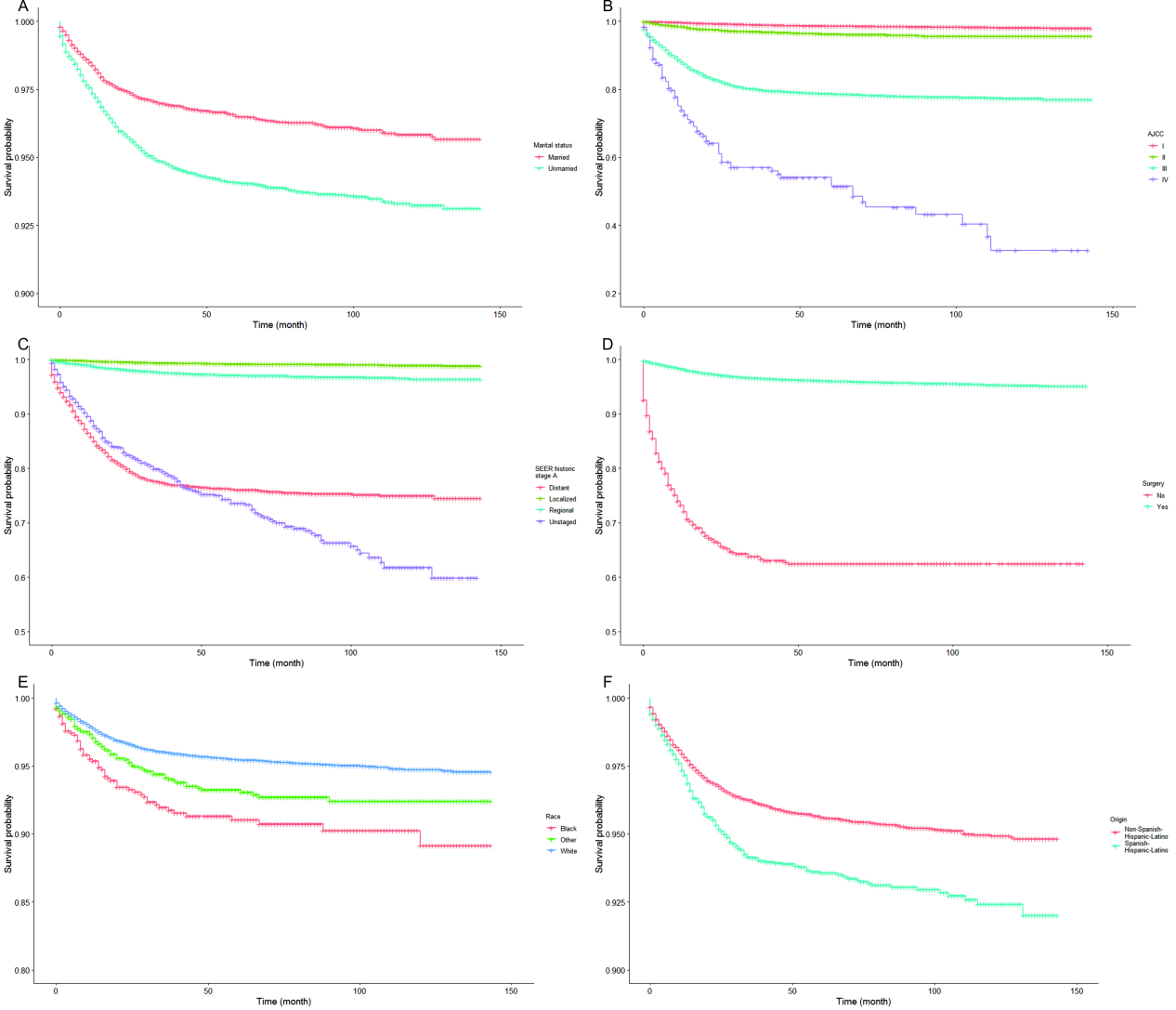


eFigure 2. Comparison chart of survival curves of testicular cancer patients. (A) Marital status: married and unmarried. (B) AJCC stage: I, II, III and IV. (C) SEER historic stage A: localized, regional, distant and unstaged. (D) Surgery: performed surgery and not performed surgery. (E) Race: black, white and others. (F) Origin: Spanish-Hispanic-Latino and Non-Spanish-Hispanic-Latino.
